# Normative Data on Inferior Mesenteric Vein Caliber in the Pediatric Population

**DOI:** 10.3390/jcm15145643

**Published:** 2026-07-18

**Authors:** Ozan Okyay, Serhat Binici, Fırat Aslan, Iklil Eryılmaz, Uğur Yanç, Veysel Tahiroğlu, Burhan Beger, Mehmet Tolgahan Örmeci, İlyas Dündar, Mahmut Baran Yerlikaya, Ali Rıza Karayıl, Orhan Beger

**Affiliations:** 1 Department of General Surgery, Van Training and Research Hospital, University of Health Sciences, 65090 Van, Türkiye; 2 Department of General Surgery, Faculty of Medicine, Van Yüzüncü Yıl University, 65090 Van, Türkiyegcbaranyerlikaya@gmail.com (M.B.Y.); 3 Department of General Surgery, Kartal Dr. Lutfi Kirdar City Hospital, 34865 İstanbul, Türkiye; 4 Department of Radiology, Şırnak State Hospital, 73000 Şırnak, Türkiye; 5 Department of Nursing, Faculty of Health Sciences, Şırnak University, 73000 Şırnak, Türkiye; 6 Department of Pediatric Surgery, Faculty of Medicine, Van Yüzüncü Yıl University, 65090 Van, Türkiye; 7 Department of Radiology, Haydarpaşa Numune Training and Research Hospital, 34668 Istanbul, Türkiye; 8 Department of Radiology, Faculty of Medicine, Van Yüzüncü Yıl University, 65090 Van, Türkiye; 9 Department of Anatomy, Faculty of Medicine, Gaziantep University, 27410 Gaziantep, Türkiye; obeger@gmail.com

**Keywords:** inferior mesenteric vein, portal system, L1, vascular disorders

## Abstract

**Objective:** The inferior mesenteric vein (IMV) is an important component of the portal venous system; however, normative developmental data during childhood remain limited. This study aimed to evaluate age- and sex-related changes in IMV caliber using contrast-enhanced computed tomography in a pediatric population. **Methods:** Radiologic images of 200 subjects aged 1–20 years were included in the examination. Terminal (IMV1), intermediate (IMV2), and pelvic (IMV3) calibers of the IMV were measured. Ratios of vessel calibers to the transverse diameter of the first lumbar vertebral body (L1TD) were calculated. In addition, the drainage pattern of the IMV was recorded. **Results:** L1TD, IMV1, IMV2, and IMV3 demonstrated significant age-related changes (*p* < 0.001). All IMV calibers increased from infancy to the postpubescent period. Among the calculated ratios, IMV1/L1TD and IMV3/L1TD demonstrated significant age-related variation (*p* < 0.05), whereas IMV2/L1TD remained relatively stable throughout childhood and adolescence (*p* > 0.05). No significant sex-related differences were observed for most parameters, although IMV3 was significantly greater in males than in females (*p* = 0.043). The splenic vein was the most common termination site of the IMV (47.5%), followed by the superior mesenteric vein (29.5%) and the splenomesenteric confluence (23.0%). **Conclusions:** The present study provides normative age-specific values for IMV caliber and IMV-to-L1TD ratios in the pediatric population. These data may facilitate the radiological assessment of IMV-related abnormalities and provide a practical reference for routine computed tomography evaluation of the pediatric mesentericoportal venous system.

## 1. Introduction

The inferior mesenteric vein (IMV) constitutes the principal venous drainage pathway of the hindgut and serves as an important component of the portal venous system. Venous blood from the distal transverse colon, descending colon, sigmoid colon, and rectum is conveyed through the IMV toward the mesentericoportal circulation. After leaving the pelvis, the superior rectal vein crosses the left common iliac vessels and continues cranially as the IMV. The vein then ascends retroperitoneally along the left side of the abdomen in close relationship to the inferior mesenteric artery (IMA), courses anterior to the psoas major muscle, and ultimately joins the portal venous system through the splenic vein (SV), the superior mesenteric vein (SMV), or the confluence of the SV and SMV [1,2]. Despite its anatomical complexity and variable termination patterns, the IMV has received considerably less attention in the literature than its arterial counterpart, the IMA [3]. Existing studies have primarily focused on its surgical significance, whereas quantitative data regarding its drainage pattern, caliber, and topographic relationships remain limited, particularly in the pediatric population [4,5,6,7]. The clinical relevance of the IMV extends beyond surgical practice, as alterations in its morphology and flow characteristics on radiologic images may provide important diagnostic clues in patients with portal venous disorders, including portal hypertension and mesenteric venous thrombosis [2,5]. Akpinar et al. [2] described marked dilatation of the IMV in a 10-year-old patient with portal vein agenesis, highlighting that clinically significant IMV abnormalities may also occur during childhood. In this regard, detailed knowledge of IMV anatomy in the pediatric population is essential for clinicians.

The caliber of the IMV may be affected by several pathological conditions and, therefore, has potential clinical significance. Le and Romanelli [8] described a patient with left colonic bleeding resulting from mesenteric varices secondary to a critical stenosis at the portal vein–IMV confluence. Ivan et al. [9] reported that patients with locally advanced rectal cancer exhibited significantly larger IMV diameters than controls and suggested that IMV caliber may serve as a marker of lymph node status, extramural venous invasion, and tumor response to chemoradiotherapy. These observations indicate that alterations in IMV caliber may reflect underlying hemodynamic and pathological processes. Therefore, accurate identification of pathological changes in the IMV requires knowledge of normal reference values. Anthropometric characteristics of individuals, such as sex, age, weight, height, body surface area, or body mass index, affect vessel calibers; thus, some researchers recommend the use of alternative anthropometric indicators, such as the body of the first lumbar vertebra (L1), to establish a definitive standardization in the diagnosis of vascular pathologies [10,11,12]. Beger and Ten [11] calculated the abdominal aorta-to-L1 ratio (the range for proximal aorta: 0.30–0.48, and the range for distal aorta: 0.23–0.38) in a pediatric reference population, and explained that a ratio above the normative range might be used as a sign of aortic dilatation, and a ratio below it might be used as a sign of aortic stenosis. Normative anatomical and morphometric data regarding the IMV in the pediatric population remain scarce in the current literature. In this regard, our retrospective study aimed to establish normative reference values for IMV caliber in a pediatric population, to determine IMV-to-L1 ratios, and to document IMV drainage patterns.

## 2. Material and Methods

### 2.1. Ethical Statement

Ethical approval was obtained from the Ethics Committee of Şırnak University to conduct this retrospective computed tomography (CT) study (approval no: 2026/168820, date: 8 May 2026).

### 2.2. Study Design

The files of patients aged 1–20 years who underwent contrast-enhanced abdominopelvic CT scans in the last 10 years (January 2016–December 2025) were accessed from the electronic hospital archive system. These files contained the following data: demographic information (sex, age), complaints (e.g., abdominal pain), diagnosis and treatment protocols (laboratory analyses, radiologic scans, clinical tests, drug information, etc.), and hospital admission and discharge dates.

An a priori power analysis was performed using G*Power software (version 3.1.9.7, Heinrich Heine University, Düsseldorf, Germany) for one-way analysis of variance (ANOVA) across 20 age groups. Based on the marked age-related differences in pediatric abdominal vascular calibers reported in previous morphometric studies [10,11], a large effect size (f = 0.50) was assumed. With α = 0.05 and power = 0.80, the minimum required total sample size was calculated as 99 subjects. To obtain balanced age- and sex-specific reference data, 10 subjects (5 males and 5 females) were included for each year of age, resulting in a final sample of 200 subjects.

### 2.3. Inclusion and Exclusion Criteria

Subjects included in the study population were selected from patients who underwent contrast-enhanced abdominopelvic CT examinations for various clinical indications, including pelvic pain, minor trauma, and abdominal pain, and were subsequently discharged without requiring medical or surgical treatment. Eligibility was determined through the evaluation of radiological findings, electronic medical records, and available clinical history. Only subjects with radiologically unremarkable abdominopelvic CT findings, no history of diseases affecting the mesentericoportal venous system, and adequate image quality were included.

Exclusion criteria were as follows: (a) genetic syndromes, systemic disorders, congenital anomalies, portal hypertension, or any condition potentially affecting mesentericoportal vascular anatomy; (b) previous abdominal, hepatobiliary, pancreatic, splenic, or cardiovascular surgery; (c) masses, cysts, lymphadenopathy, vascular abnormalities, or other pathological findings involving the abdominopelvic organs; and (d) hemorrhage, free fluid, free air, or radiological evidence of abdominal trauma.

### 2.4. Study Population

The 200 subjects (mean age: 10.50 ± 5.78 years; 100 males, 100 females) were included in this retrospective CT examination.

### 2.5. Computed Tomography Protocol

A multidetector CT scanner was used to obtain the contrast-enhanced abdominopelvic CT images (Toshiba Alexion, TSX-034A, Tochigi, Japan). Patients were asked to fast for 4–6 h before the scan. In the majority of patients (over 6 years of age), the scans were carried out in the inspiratory phase. In some pediatric cases under 6 years of age, sedation was applied, and the scans were carried out during free breathing. While the scans were being carried out, “as low as reasonably achievable” (ALARA) guidelines were taken into consideration; thus, appropriate milliampere-second (mAs) and peak kilovoltage (kVp) values were determined for each patient. The scan parameters were determined as follows: image matrix = 512 × 512, beam pitch = 1.00, field of view (FOV) = 30–40 cm, slice thickness = 1 mm, effective mAs = 80, and tube voltage = 80–120 kVp (variable). A standard radiologic protocol was followed, and each patient received an intravenous non-ionic iodinated contrast agent, followed by physiological saline (between 20 and 30 mL). Contrast agent (iohexol, 350 mg/mL; GE Healthcare, Cork, Ireland) was administered at a rate of 2–4 mL/s. A standard volume of 70 mL was used, with weight-based dose adjustment in children (1–2 mL/kg) and a maximum total volume of 80 mL. Axial CT views were acquired, and then sagittal, coronal, and three-dimensional reformatted views were reconstructed. The data were transferred to the radiological imaging system in the hospital (picture archiving and communication system, PACS) and then analyzed via the Sectra Workstation IDS7 software (version 26.2; Sectra, Linköping, Sweden).

### 2.6. Morphometric Parameters

**Figure 1:**
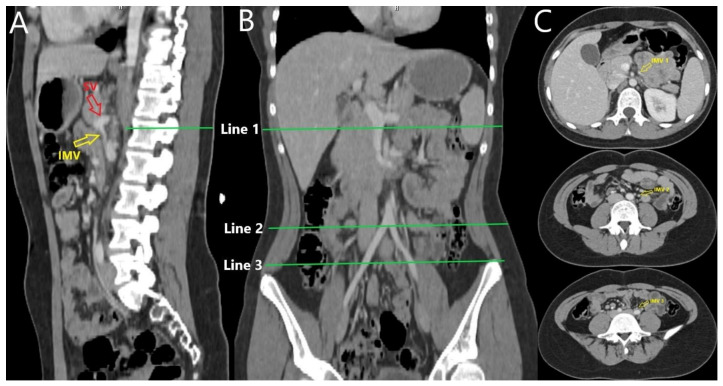
Measurement levels of the inferior mesenteric vein (IMV) in a 14-year-old girl. (**A**) Sagittal computed tomography (CT) image demonstrating the termination of the IMV into the splenic vein (SV). (**B**) Coronal reconstruction showing the three predefined measurement levels: IMV1 (terminal segment, line 1), IMV2 (aortic bifurcation level, line 2), and IMV3 (pelvic brim level, line 3). (**C**) Corresponding axial CT images demonstrating the measurement of IMV caliber at each level. IMV diameters were measured perpendicular to the long axis of the vein from outer wall to outer wall.

IMV diameter was measured at three different levels (Figure 1). The first IMV caliber (IMV1) was measured 3 mm proximal to the venous termination site. The second IMV caliber (IMV2) was measured at the level of the aortic bifurcation. The third IMV caliber (IMV3) was measured at the level where the vein crosses the iliac vessels while entering the lesser pelvis (i.e., at the level of the pelvic brim). Oblique multiplanar reconstructions were created to measure IMV1, IMV2 and IMV3 on axial CT images perpendicular to IMV’s long axis. IMV1, IMV2, and IMV3 were measured using the distances from one outer wall of the vein to the other [13].

**Figure 2:**
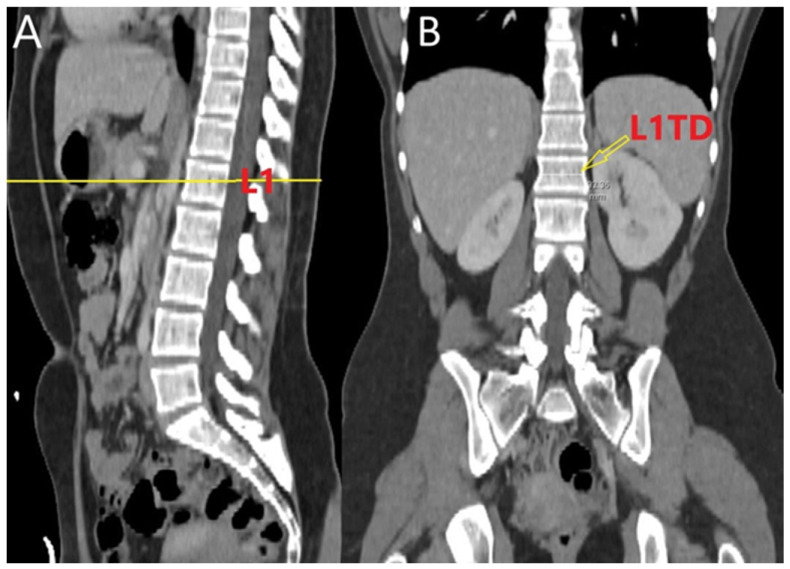
The first lumbar vertebra (L1) and its diameter in a 14-year-old girl. (**A**) The location of L1 on the sagittal section. (**B**) The transverse caliber of L1’s body at its center (L1TD) was measured.

L1 was chosen as the anthropometric indicator in the current study; thus, we measured the transverse diameter of the L1 (L1TD) (Figure 2) [13]. The ratios of IMV1, IMV2, and IMV3 to L1TD were calculated.

### 2.7. Termination Patterns of the Inferior Mesenteric Vein

The termination site of the IMV was recorded in all subjects.

### 2.8. Statistical Analysis

An independent radiologist (U.Y.) with seven years of experience performed all measurements, and these measurements were used for the statistical analyses. To assess reproducibility, a second radiologist (M.T.Ö.), also with seven years of experience, independently measured all parameters twice in 40 randomly selected subjects (20% of the study population). The first set of measurements obtained by the second radiologist was compared with the measurements of the first radiologist to assess inter-observer reproducibility, whereas the two measurements obtained by the second radiologist were compared to assess intra-observer reproducibility. Intra-class correlation coefficients (ICCs) were used to assess inter-observer reproducibility, and the paired-samples t-test was used to assess intra-observer reproducibility. Sex comparisons were made using independent-samples *t*-tests. Alterations in the parameters according to age (1–20 years) were analyzed using one-way ANOVA. In light of Goodway et al.’s book [14], pediatric patients were divided into five age groups as follows: infancy: 1–2 years, early childhood: 3–5 years, late childhood: 6–9 years, prepubescent: 10–13 years, and postpubescent: 14–20 years. Changes in the parameters according to age groups (from the infancy period to the postpubescent period) were analyzed using one-way ANOVA. Following a significant omnibus one-way ANOVA, Bonferroni-adjusted pairwise comparisons were performed among the five pediatric age groups. Comparisons of IMV1, IMV2, and IMV3 were performed using repeated-measures ANOVA. Correlations between IMV-related parameters were assessed using the Pearson correlation coefficient test. Associations between sex and IMV termination patterns were evaluated using the Pearson chi-square test. Curvilinear or linear regression analysis was utilized to reveal growth dynamics of IMV-related parameters in relation to age. The coefficient of determination (R^2^) was used to assess the match between the estimated functions and the numerical values. Levene’s test was used to evaluate homoscedasticity. The normality control of the dataset was made using the Shapiro–Wilk test. This test was performed after dividing the study population into age groups. Parametric tests were used in statistical analyses, since the data were normally distributed. Using SPSS version 25.0 (IBM, Armonk, NY, USA), all analyses were performed by accepting “*p* < 0.05” as significant.

## 3. Results

The intra-observer (*p* > 0.05) and inter-observer (ICC scores of 0.94–0.98 for the parameters; *p* < 0.001) assessments indicated good repeatability of measurements.

Age-specific measurements are presented in Table 1. Age significantly influenced L1TD and all three IMV calibers (*p* < 0.001), as well as IMV1/L1TD and IMV3/L1TD (*p* = 0.001); IMV2/L1TD did not vary significantly with age (*p* = 0.116).

According to pediatric age groups (Table 2), L1TD increased progressively from infancy to the postpubescent period (*p* < 0.001). Similarly, IMV1, IMV2, and IMV3 demonstrated a gradual increase across age groups, reaching their highest values during the postpubescent period (*p* < 0.001). Among the calculated ratios, IMV1/L1TD and IMV3/L1TD showed significant differences according to age and age groups (*p* < 0.05). In contrast, IMV2/L1TD remained relatively stable throughout childhood and adolescence and did not demonstrate significant age-related variation (*p* > 0.05).

Comparison of the parameters according to sex is presented in Table 3. Sex had no significant effect on L1TD, IMV1, IMV2, IMV1/L1TD, IMV2/L1TD, or IMV3/L1TD (*p* > 0.05). However, IMV3 was significantly greater in males than in females (*p* = 0.043).

Significant positive correlations were observed among all IMV calibers and L1TD (*p* < 0.001). L1TD demonstrated moderate-to-strong positive correlations with IMV1 (*r* = 0.519), IMV2 (*r* = 0.685), and IMV3 (*r* = 0.706). In addition, strong positive correlations were identified among the IMV calibers themselves (*p* < 0.001). IMV1 was strongly correlated with IMV2 (*r* = 0.830) and IMV3 (*r* = 0.672), whereas the strongest correlation was observed between IMV2 and IMV3 (*r* = 0.910).

IMV1 (5.22 ± 1.40 mm) was significantly greater than IMV2 (4.57 ± 1.36 mm) and IMV3 (4.11 ± 1.49 mm), while IMV2 was significantly greater than IMV3 (*p* < 0.001 for all comparisons). These findings indicate a progressive increase in IMV caliber from the pelvic brim level to the termination site.

The termination pattern of the IMV was documented in all subjects. The most common drainage site was the SV (95/200, 47.5%), followed by the SMV (59/200, 29.5%) and the splenomesenteric confluence (46/200, 23.0%). Analysis of IMV termination patterns according to sex demonstrated no significant association between sex and the site of venous termination (χ^2^ = 3.537, *p* = 0.171). In males, the IMV drained into the SV in 46.0% of cases, the SMV in 35.0%, and the splenomesenteric confluence in 19.0%. In females, the corresponding frequencies were 49.0%, 24.0%, and 27.0%, respectively.

**Figure 3:**
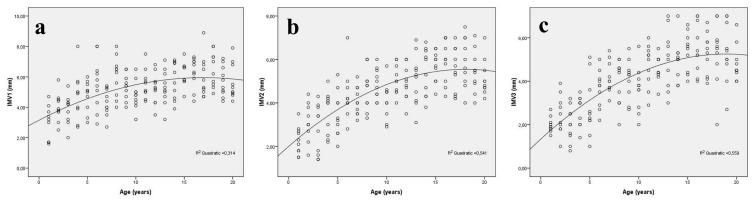
Quadratic functions. (**a**) terminal inferior mesenteric vein caliber (IMV1), (**b**) intermediate inferior mesenteric vein caliber (IMV2), and (**c**) pelvic inferior mesenteric vein caliber (IMV3).

Quadratic functions were calculated for the parameters as follows (Figure 3):

- IMV1 = 3.153 + 0.330 × (age) − 0.010 × (age)^2^ (R^2^: 0.314, *p* < 0.001)
- IMV2 = 2.004 + 0.393 × (age) − 0.011 × (age)^2^ (R^2^: 0.541, *p* < 0.001)
- IMV3 = 1.337 + 0.414 × (age) − 0.011 × (age)^2^ (R^2^: 0.559, *p* < 0.001)

## 4. Discussion

To our knowledge, this is the first study to report age- and sex-specific IMV calibers at the pelvic, intermediate, and terminal levels, together with the corresponding IMV1/L1TD, IMV2/L1TD, and IMV3/L1TD ratios, in a pediatric reference population aged 1–20 years. These data provide morphometric reference standards for assessing IMV-related abnormalities, including dilatation.

Accurate assessment of vascular dilatation or narrowing is important for radiologists and surgeons and requires appropriate reference values [10,11,12]. Although an arterial diameter exceeding 1.5 times its expected normal value is commonly considered dilatation [15,16], fixed thresholds should be interpreted cautiously because vascular calibers vary according to demographic and anthropometric characteristics [10,12]. Accordingly, indicators such as body surface area have been used to normalize vascular measurements [17]. For example, abdominal aortic and main portal vein calibers correlate positively with age, height, weight, and body surface area [10,17]. However, reliable anthropometric measurements may be unavailable or difficult to obtain in emergency settings, particularly in infants and young children [18]. L1TD has therefore been proposed as an internal anatomical indicator for relating vessel caliber to body size [10,11,12]. L1 is easily identified and visible on almost all abdominopelvic CT examinations, while minor variations in its orientation have little effect on diameter or area measurements. Furthermore, L1-based indices account for body habitus in cross-sectional imaging [19]. Evidence supporting L1-based anatomical normalization in vascular morphometry has been reported previously [11,12,13]. Aktürk and Gunes [12] used L1TD to standardize pediatric abdominal aortic measurements, particularly when anthropometric indicators were unavailable or difficult to obtain. Beger and Ten [11] likewise reported age-dependent changes in pediatric abdominal visceral artery-to-L1 ratios. Binici et al. [13] reported that age-specific portal vein-to-L1 ratios in subjects aged 1–80 years may provide reference data for evaluating portal venous disorders. The broader concept of vertebral body–based normalization has also been examined outside the abdomen. Akay et al. [20] found that ratios between thoracic vascular diameters and the anteroposterior diameter of a thoracic vertebral body remained relatively stable across pediatric age groups despite increases in absolute vessel calibers, supporting vertebral measurements as internal anatomical references [20]. The present study applies L1-based normalization to the mesentericoportal venous system and provides age-specific IMV-to-L1TD reference data for assessing vascular abnormalities. Because vertebral body size reflects body habitus and skeletal growth [19], L1TD provides an internal normalization parameter that can be directly measured on the same CT examination when conventional anthropometric data are unavailable or incomplete, particularly in emergency settings [13,18]. Therefore, L1-based normalization should complement, rather than replace, conventional anthropometric parameters.

Several pathological conditions may substantially affect IMV caliber. Prasad et al. [21] described a 4-year-old girl with extrahepatic portal vein obstruction who presented with chronic anemia and melena. Surgical exploration demonstrated a giant IMV measuring more than 3 cm in diameter, accompanied by colonic varices. Khan et al. [22] reported a 10-year-old boy with Abernethy malformation who presented with intermittent abdominal pain and hematochezia. Contrast-enhanced CT demonstrated a markedly dilated IMV associated with a direct fistulous communication between the IMV and the internal iliac vein. These observations indicate that significant alterations in IMV caliber may occur in pediatric patients with portal venous and portosystemic vascular disorders [21,22]. Furthermore, Zhang and Li [23] reported a 9-year-old girl with portal hypertension secondary to congenital absence of the right portal vein who was successfully treated with a proximal IMV-to-left renal vein shunt. According to Deshmukh et al. [24], the inherently smaller caliber of the IMV may represent an advantage during portosystemic shunt procedures because it is less likely to divert the entire portal blood flow away from the liver. These findings suggest that IMV caliber may have not only diagnostic but also surgical and hemodynamic significance [23,24]. Accordingly, accurate IMV identification and knowledge of age-appropriate caliber ranges may aid the evaluation and management of selected pediatric portal venous disorders; however, the clinical utility of the age-specific reference values and regression equations reported here requires validation in future studies involving patients with mesentericoportal disorders.

The mean IMV1, IMV2, and IMV3 calibers were 5.22 ± 1.40 mm (range: 1.60–8.90 mm), 4.57 ± 1.36 mm (range: 1.40–7.50 mm), and 4.11 ± 1.49 mm (range: 0.80–7.00 mm), respectively. All three calibers increased across age groups and reached their highest values during the postpubescent period. Most parameters showed no sex-related differences, although IMV3 was greater in males. Comparable pediatric data are unavailable, and quantitative adult data remain limited. In 35 formalin-fixed adult cadavers, Le et al. [25] reported mean IMV diameters of 4.8 ± 0.9 mm at the confluence of the superior rectal veins and 6.3 ± 1.1 mm at the portal termination. Although these sites approximately correspond to IMV3 and IMV1, direct comparison is limited by differences in age, specimen type, and measurement methodology. In our cohort, IMV caliber increased from the pelvic brim to the termination site, demonstrating its noncylindrical morphology. IMV caliber should be interpreted at defined segment-specific levels rather than using a single cutoff. The mean IMV1/L1TD, IMV2/L1TD, and IMV3/L1TD ratios were 0.14 ± 0.04 (range: 0.07–0.26), 0.12 ± 0.03 (range: 0.05–0.22), and 0.11 ± 0.03 (range: 0.03–0.18), respectively. IMV1/L1TD and IMV3/L1TD varied significantly with age, whereas IMV2/L1TD remained relatively stable throughout childhood and adolescence; none of the ratios differed significantly by sex. Similar L1-based approaches have been applied to other abdominal vessels. Aktürk and Gunes [12] reported a proximal abdominal aorta-to-L1 ratio of 0.41 in pediatric subjects younger than 133 months, in whom L1TD did not differ by sex, and suggested that values exceeding 0.4 might indicate aortic dilatation. Ten and Beger [10] reported a proximal IMA-to-L1 ratio of 0.08 in children aged 1–18 years. These examples demonstrate the potential value of vessel-to-L1 ratios in segment-specific vascular assessment. Accordingly, values outside the reported segment-specific ranges may help identify potentially abnormal IMV calibers, including segmental dilatation. Because L1 is readily visualized on abdominopelvic CT, including IMV-to-L1 ratios alongside IMV measurements in routine reports may provide a practical adjunct to radiological assessment [13]. Further studies in patients with different mesentericoportal disorders are required to clarify their clinical relevance and establish validated diagnostic thresholds.

In our pediatric cohort, the IMV terminated most often in the SV (47.5%), followed by the SMV (29.5%) and the splenomesenteric confluence (23.0%). Adult radiological and anatomical studies have shown substantial variation. Using helical CT venography in 54 adults, Graf et al. [26] reported termination in the SV, SMV, and confluence in 56%, 26%, and 18%, respectively. In 916 adults examined with multidetector-row CT, Krumm et al. [27] reported corresponding rates of 37.6%, 19.2%, and 28.8%, with uncommon variants in 14.4%. Using three-dimensional CT angiography in 167 patients undergoing laparoscopic surgery for left-sided colorectal cancer, Nepal et al. [7] reported rates of 50%, 26%, and 22%, respectively, with middle colic vein drainage in 2%. Preoperative three-dimensional CT in 66 patients with colorectal cancer yielded corresponding rates of 48.5%, 40.9%, and 10.6% in the study by Arimoto et al. [28]. In contrast, Yılmaz et al. [29] identified the SMV as the most frequent termination site in 877 multidetector CT examinations (45.4%), followed by the SV (43.2%) and the confluence (8.1%); the remaining variants were uncommon. Despite differences in study populations, imaging methods, and classification systems, these findings establish the SV, SMV, and splenomesenteric confluence as the principal IMV termination sites, with our pediatric distribution broadly comparable to adult data.

This study has several limitations. First, the sample size was moderate; larger cohorts are needed to characterize age-related changes in IMV caliber more precisely. Second, L1TD was the only body-size indicator because the retrospective records did not consistently provide height, weight, or body mass index. Future retrospective or prospective studies incorporating body surface area and other anthropometric measures could compare normalization approaches and better characterize IMV growth during childhood. Third, measurements were obtained from two-dimensional CT images, and three-dimensional methods may provide additional morphometric information. Fourth, the analyses were based on radiological reconstructions rather than anatomical specimens; therefore, image resolution and software-related processing may have affected linear measurements, particularly in younger children. Finally, this hospital-based CT study included patients examined for clinical indications rather than healthy volunteers, although none had detectable abdominopelvic pathology. Residual selection bias and undetected subclinical conditions, therefore, cannot be excluded. Because CT performed solely for research cannot be ethically justified in healthy children, the findings should be interpreted as CT-based reference data.

## 5. Conclusions

Measurements of diameter by age, regression equations, and variations related to IMV may provide useful information to clinicians in diagnosing IMV-related abnormalities in children. This may also aid in planning preoperative strategies for surgeries, such as those for IMV dilatation. We suggest that the IMV-to-L1 ratio may be added to routine CT reports for detecting conditions like IMV aneurysms, considering that L1 is easily identifiable in abdominopelvic imaging. Further research to assess this ratio in various diseases and to establish precise threshold values would enhance understanding of its clinical relevance.

## Figures and Tables

**Table 1:** Age-dependent changes in the parameters from the age of 1 to 20 years.

Age	L1TD (mm)	IMV1 (mm)	IMV2 (mm)	IMV3 (mm)	IMV1/L1TD	IMV2/L1TD	IMV3/L1TD
1	22.29 ± 0.66	2.88 ± 1.14	2.26 ± 0.64	1.98 ± 0.35	0.13 ± 0.05	0.10 ± 0.03	0.09 ± 0.02
2	24.69 ± 1.76	3.99 ± 0.91	3.11 ± 0.84	2.44 ± 0.88	0.16 ± 0.04	0.13 ± 0.04	0.10 ± 0.04
3	26.96 ± 1.20	3.88 ± 0.95	2.77 ± 1.13	2.01 ± 0.80	0.15 ± 0.04	0.10 ± 0.04	0.08 ± 0.03
4	27.13 ± 1.87	4.76 ± 1.55	3.56 ± 0.98	2.66 ± 0.69	0.17 ± 0.05	0.13 ± 0.03	0.10 ± 0.02
5	28.65 ± 2.26	4.74 ± 1.00	3.47 ± 1.02	2.78 ± 1.33	0.17 ± 0.04	0.12 ± 0.03	0.10 ± 0.04
6	31.82 ± 1.09	5.56 ± 1.71	4.34 ± 1.11	3.65 ± 0.85	0.17 ± 0.05	0.14 ± 0.03	0.11 ± 0.03
7	32.30 ± 2.24	4.30 ± 0.87	4.09 ± 0.64	4.16 ± 0.73	0.13 ± 0.03	0.13 ± 0.03	0.13 ± 0.02
8	35.24 ± 2.70	6.03 ± 1.31	4.64 ± 0.94	3.96 ± 1.02	0.17 ± 0.04	0.13 ± 0.03	0.11 ± 0.03
9	37.30 ± 2.09	5.34 ± 0.84	4.88 ± 0.69	4.10 ± 0.89	0.14 ± 0.02	0.13 ± 0.02	0.11 ± 0.02
10	38.28 ± 2.50	4.81 ± 0.92	4.12 ± 0.84	3.62 ± 1.02	0.13 ± 0.02	0.11 ± 0.02	0.09 ± 0.03
11	38.78 ± 3.72	5.52 ± 0.95	5.06 ± 0.68	4.74 ± 0.98	0.14 ± 0.03	0.13 ± 0.02	0.12 ± 0.03
12	40.16 ± 2.13	5.23 ± 1.01	4.92 ± 0.79	4.56 ± 0.59	0.13 ± 0.03	0.12 ± 0.02	0.11 ± 0.01
13	43.22 ± 2.15	5.30 ± 1.13	5.07 ± 1.17	5.02 ± 1.34	0.12 ± 0.03	0.12 ± 0.03	0.12 ± 0.03
14	42.07 ± 3.36	5.78 ± 1.22	5.42 ± 1.08	4.96 ± 0.92	0.14 ± 0.03	0.13 ± 0.03	0.12 ± 0.02
15	43.55 ± 1.84	6.18 ± 0.79	5.69 ± 0.70	5.37 ± 0.89	0.14 ± 0.02	0.13 ± 0.02	0.12 ± 0.02
16	43.12 ± 3.06	6.29 ± 1.00	5.93 ± 0.99	5.53 ± 1.14	0.15 ± 0.03	0.14 ± 0.03	0.13 ± 0.03
17	45.48 ± 3.93	5.99 ± 1.33	5.53 ± 1.00	5.26 ± 0.95	0.13 ± 0.03	0.12 ± 0.02	0.12 ± 0.02
18	45.52 ± 2.47	6.52 ± 1.14	5.88 ± 1.12	5.30 ± 1.46	0.14 ± 0.02	0.13 ± 0.03	0.12 ± 0.03
19	46.04 ± 2.64	5.71 ± 0.99	5.44 ± 0.95	5.31 ± 1.36	0.12 ± 0.02	0.12 ± 0.02	0.12 ± 0.03
20	45.42 ± 4.53	5.66 ± 1.16	5.14 ± 0.83	4.81 ± 0.82	0.13 ± 0.03	0.11 ± 0.03	0.11 ± 0.03
Total	36.90 ± 7.97	5.22 ± 1.40	4.57 ± 1.36	4.11 ± 1.49	0.14 ± 0.04	0.12 ± 0.03	0.11 ± 0.03
*p*	<0.001	<0.001	<0.001	<0.001	0.001	0.116	0.001

Data are presented as mean ± standard deviation. IMV: inferior mesenteric vein; L1TD: transverse diameter of the first lumbar vertebral body; IMV1: IMV caliber measured 3 mm proximal to the venous termination site; IMV2: IMV caliber measured at the level of the aortic bifurcation; IMV3: IMV caliber measured at the level where the vein crosses the iliac vessels at the pelvic brim. IMV1/L1TD, IMV2/L1TD, and IMV3/L1TD represent the ratios between IMV calibers and L1TD. The *p*-values indicate comparisons among age groups.

**Table 2:** Comparison of first lumbar vertebral body transverse diameter (L1TD), inferior mesenteric vein (IMV) calibers, and IMV-to-L1TD ratios among pediatric age groups.

Age Groups	Infancy (*n* = 20)	Early Childhood (*n* = 30)	Late Childhood (*n* = 40)	Prepubescent (*n* = 40)	Postpubescent (*n* = 70)	*p*-Value
L1TD (mm)	23.49 ± 1.78 ^a,b,c,d^	27.58 ± 1.93 ^b,c,d^	34.17 ± 3.04 ^c,d^	40.11 ± 3.25 ^d^	44.46 ± 3.40	<0.001
IMV1 (mm)	3.44 ± 1.15 ^a,b,c,d^	4.46 ± 1.23 ^b,c,d^	5.31 ± 1.35 ^d^	5.22 ± 1.00 ^d^	6.02 ± 1.10	<0.001
IMV2 (mm)	2.69 ± 0.85 ^b,c,d^	3.27 ± 1.07 ^b,c,d^	4.49 ± 0.89 ^d^	4.79 ± 0.94 ^d^	5.58 ± 0.95	<0.001
IMV3 (mm)	2.21 ± 0.69 ^b,c,d^	2.48 ± 1.01 ^b,c,d^	3.97 ± 0.87 ^d^	4.49 ± 1.11 ^d^	5.22 ± 1.08	<0.001
IMV1/L1TD	0.15 ± 0.05	0.16 ± 0.04 ^c,d^	0.16 ± 0.04 ^c,d^	0.13 ± 0.03	0.14 ± 0.03	<0.001
IMV2/L1TD	0.11 ± 0.03	0.12 ± 0.04	0.13 ± 0.03	0.12 ± 0.02	0.13 ± 0.02	0.088
IMV3/L1TD	0.09 ± 0.03 ^b,c,d^	0.09 ± 0.03 ^b,c,d^	0.12 ± 0.03	0.11 ± 0.03	0.12 ± 0.03	<0.001

Data are presented as mean ± standard deviation. The “*n*” indicates the number of subjects. IMV: inferior mesenteric vein; L1TD: transverse diameter of the first lumbar vertebral body; IMV1: caliber measured 3 mm proximal to the IMV termination site; IMV2: caliber measured at the level of the aortic bifurcation; IMV3: caliber measured at the level where the IMV crosses the iliac vessels at the pelvic brim. IMV1/L1TD, IMV2/L1TD, and IMV3/L1TD represent the corresponding caliber-to-L1TD ratios. Superscript letters indicate statistically significant pairwise differences based on Bonferroni-adjusted post hoc comparisons: ^a^, comparison with early childhood; ^b^, comparison with late childhood; ^c^, comparison with prepubescent subjects; ^d^, comparison with postpubescent subjects (adjusted *p* < 0.05).

**Table 3:** Comparison of the parameters according to sex.

Parameters	Males (*n* = 100)	Females (*n* = 100)	*p*-Value
L1TD (mm)	37.83 ± 8.38	35.97 ± 7.47	0.100
IMV1 (mm)	5.37 ± 1.36	5.07 ± 1.42	0.128
IMV2 (mm)	4.72 ± 1.25	4.42 ± 1.46	0.123
IMV3 (mm)	4.32 ± 1.44	3.90 ± 1.51	0.043
IMV1/L1TD	0.15 ± 0.04	0.14 ± 0.04	0.598
IMV2/L1TD	0.13 ± 0.02	0.12 ± 0.03	0.440
IMV3/L1TD	0.11 ± 0.03	0.11 ± 0.03	0.137

Data are presented as mean ± standard deviation. The “*n*” indicates the number of subjects. IMV: inferior mesenteric vein; L1TD: transverse diameter of the first lumbar vertebral body; IMV1: caliber measured 3 mm proximal to the IMV termination site; IMV2: caliber measured at the level of the aortic bifurcation; IMV3: caliber measured at the level where the IMV crosses the iliac vessels at the pelvic brim. IMV1/L1TD, IMV2/L1TD, and IMV3/L1TD represent the corresponding caliber-to-L1TD ratios.

## Data Availability

Available from the corresponding author on reasonable request.

## Abbreviations

ALARA	As Low As Reasonably Achievable
ANOVA	Analysis of Variance
CT	Computed Tomography
FOV	Field of View
ICC	Intraclass Correlation Coefficient
IMA	Inferior Mesenteric Artery
IMV	Inferior Mesenteric Vein
L1	First Lumbar Vertebra
L1TD	Transverse Diameter of the First Lumbar Vertebral Body
PACS	Picture Archiving and Communication System
SMV	Superior Mesenteric Vein
SV	Splenic Vein
